# Establishment, characterization, and validation of novel porcine embryonic fibroblasts as a potential source for genetic modification

**DOI:** 10.3389/fcell.2022.1059710

**Published:** 2022-11-10

**Authors:** Chi-Hun Park, Young-Hee Jeoung, Luhui Zhang, Sai Goutham Reddy Yeddula, Ki-Eun Park, Jerel Waters, Bhanu P. Telugu

**Affiliations:** ^1^ Division of Animal Sciences, University of Missouri, Columbia, MO, United States; ^2^ RenOVAte Biosciences Inc., Reisterstown, MD, United States

**Keywords:** embryonic fibroblast, livestock, somatic cell nuclear transfer (SCNT), genome editing and engineering, embryo development

## Abstract

Fibroblasts are the common cell type in the connective tissue-the most abundant tissue type in the body. Fibroblasts are widely used for cell culture, for the generation of induced pluripotent stem cells (iPSCs), and as nuclear donors for somatic cell nuclear transfer (SCNT). We report for the first time, the derivation of embryonic fibroblasts (EFs) from porcine embryonic outgrowths, which share similarities in morphology, culture characteristics, molecular markers, and transcriptional profile to fetal fibroblasts (FFs). We demonstrated the efficient use of EFs as nuclear donors in SCNT, for enhanced post-blastocyst development, implantation, and pregnancy outcomes. We further validated EFs as a source for CRISPR/Cas genome editing with overall editing frequencies comparable to that of FFs. Taken together, we established an alternative and efficient pipeline for genome editing and for the generation of genetically engineered animals.

## Introduction

The recent discovery and successful deployment of CRISPR/Cas -based genome editing represents a significant milestone for the large animal transgenesis field (Whitelaw et al., 2016; Perisse et al., 2020; Whitworth et al., 2022), ever since the successful generation of “dolly” by somatic cell nuclear transfer (SCNT) over 2 decades ago (Campbell et al., 1996). The CRISPR reagents can be delivered directly into the zygotes (zygotic injection) or into somatic cells followed by SCNT for the generation of genome-edited (GE) piglets (Whitelaw et al., 2016; Whitworth et al., 2022). The key advantages of zygote injection approach are the high efficiency of introducing targeted genetic mutations, high pregnancy, and litter rates. However, the editing outcomes in the resulting offspring are often unpredictable, with mosaicism and off-targeting being primary concerns (Tanihara et al., 2019; Rubinstein et al., 2021). This often precludes the use of G0 founders in the phenotypic analysis, necessitating outcrossing of the founders to parse out the alleles and subsequent breeding of the offspring to homozygosity, which takes nearly 3 years in pigs. Needless to say, this strategy puts a huge strain on resources (physical space, labor, feed costs, *etc.*) to generate GE models. An alternative to zygotic injections is SCNT, which is widely used for generating GE pigs. The key advantage of SCNT is the generation of a cohort of animals with pre-determined modifications following a round of embryo transfer, and therefore a more conducive timeline for experiments. The main cell type used for genetic modification and for SCNT are the fibroblasts, which represent the most abundant cell type in the connective tissue of the body and are involved in the maintenance of structural integrity and architecture of tissues and organs. Fibroblasts can be easily isolated from diverse tissue sources and maintained under standard culture conditions, and hence are used extensively for cell culture and cellular reprogramming (e.g., SCNT and iPSCs). Even though adult tissue-derived fibroblasts represent an easily accessible cell source, they have a limited life span in culture and can present a significant challenge for long-term culture to screen for targeted mutations in clonal lines (Park et al., 2021). Thus, researchers prefer using fibroblasts of fetal origin with a relatively extended proliferative capacity.

Despite significant efforts for improving the SCNT process, including the choice of somatic cell source (Inoue et al., 2003; Wang et al., 2020), success in generating normal viable offspring by SCNT remains a formidable challenge with efficiencies ranging from 1 to 5%, and in desperate need of improvement (Keefer, 2015; Matoba and Zhang, 2018). The process of SCNT can be impacted by numerous factors, which makes it extremely difficult to correlate the developmental defects in cloned embryos (Inoue et al., 2003; Gouveia et al., 2020). Adding to this difficulty is the poor viability of fibroblasts following cell-mediated transgenesis, which can adversely impact embryonic development (Matoba and Zhang, 2018). Based on the evidence that the tissue-specific cells can be derived by directed differentiation of human and mouse pluripotent stem/precursor cells (Torres et al., 2012; Bao et al., 2016; D'Angelo et al., 2018; Wang et al., 2020), we sought to develop a method for generating primary fibroblast-like cells from pluripotent cells of embryonic outgrowths, and test the hypothesis that the primary cells derived from the embryo serve as an ideal nuclear donor, and result in high SCNT efficiencies. The rationale is based on previously reported finding that genetically modified FFs that hitherto failed to generate a viable offspring by SCNT were able to give rise to embryo-derived extraembryonic endoderm stem cells (XEN), and the use of rederived embryonic cells resulted in viable cloned offspring with high SCNT efficiencies (Park et al., 2021).

In this report, we demonstrate the successful and efficient derivation of embryo-derived fibroblasts (EFs), which share many characteristics with fetal fibroblasts (FFs), including the spindle-shaped morphology and expression of key fibroblast-specific molecular markers. In addition, transcriptomic analysis *via* RNA sequencing (RNA-seq) further confirmed the close similarity of EF to FF. The EFs used as nuclear donors for SCNT resulted in enhanced *in vivo* developmental competence. Finally, we demonstrated the feasibility of establishing EFs from edited embryos and the feasibility of genetic modification directly in the EFs. The ability to pre-screen the clonal EF lines without the need for performing embryo transfers to establish FF lines is expected to overcome the current limitation of SCNT (poor reprogramming efficiency with the use of FF lines) and zygotic microinjection (unpredictability of edited outcomes and mosaicism). The established EFs may, therefore, represent an important cell resource for gene-editing and for generating genetically modified animals.

## Materials and methods

All chemicals were purchased from Sigma-Aldrich (St. Louis, MO) unless stated otherwise. All experiments involving live animals were performed as per the approved guidelines of the University of Missouri, Institutional Animal Care and Use Committee protocol# 14400.

### Generation of *in vitro*, *in vivo,* and SCNT pig embryos

Porcine *in vitro*, *in vivo,* and *SCNT* embryos were produced as described in our previous studies (Park et al., 2021). Briefly, cumulus-oocyte-complexes (COC) were purchased from a commercial supplier (De Soto Biosciences, Seymour, TN, United States). After *in vitro* maturation, the cumulus cells were removed from oocytes by gentle pipetting in a 0.1% (w/v) hyaluronidase solution. For *in vitro* fertilization (IVF), pre-diluted fresh semen (commercially sourced from PIC) was centrifuged twice at 200 g for 3 min in DPBS containing 0.2% BSA. The sperm pellet was adjusted to a concentration of 2 × 10^5^ sperm per mL and co-incubated with matured oocytes in a modified Tris-buffered medium containing 0.4% BSA for 5 h in a humidified atmosphere (5% CO_2_ in air). For SCNT, embryonic or fetal fibroblasts were synchronized to the G1/G0-phase by serum deprivation (DMEM with 0.1% FBS) for 96 h. The oocytes are enucleated by aspirating the polar body and the MII metaphase plates by a micropipette (Humagen, Charlottesville, VA, United States) in 0.1% DPBS supplemented with 5 μg/ml of cytochalasin B. After enucleation, a donor cell was placed into the perivitelline space of an enucleated oocyte. The cell–oocyte couplets were fused by applying two direct current (DC) pulses (1-s interval) of 2.0 kV/cm for 30 μs using an ECM 2001 Electroporation System (BTX, Holliston, MA). After fusion, the reconstructed oocytes were activated by a DC pulse of 1.0 kV/cm for 60 μs, followed by post-activation in 2 mM 6-dimethylaminopurine for 3 h. After overnight culture in PZM3 with a histone deacetylase inhibitor Scriptaid (0.5 μM), the cloned embryos were cultured and maintained in PZM3 medium in a low oxygen environment (5% O_2_, 5% CO_2_ and 90% N_2_). Embryos were transferred into synchronized recipients on the first day of standing estrus. Pregnancies were confirmed by ultrasound on day 25–27 following embryo transfer and fetuses were retrieved around day 40 of pregnancy.

### Establishment and culture of adult, fetal and embryonic fibroblasts

Adult fibroblast (AF) cells were obtained from ear skin biopsies, and fetal fibroblast (FF) cells were obtained from fetuses from artificially inseminated embryos on day 28 of pregnancy. The tissue samples were cut into small pieces (∼1 mm^3^). Dissected tissues were then cultured in Dulbecco’s modified Eagle’s medium (DMEM; Gibco-BRL) supplemented with 10% fetal bovine serum (FBS; Atlanta biologicals), 1 mM sodium pyruvate, 2 mM L-glutamine, penicillin-streptomycin, 0.1 mM 2-β-mercaptoethanol, 1% non-essential amino acids (NEAA), 100 units/mL antibiotics and antimycotics until reaching confluency at 38.5°C in 5% CO_2_ and air. For establishing EFs, primary outgrowths from d7 embryos were established as previously described (Park et al., 2021). Briefly, expanded blastocysts either fully hatched or zona denuded by brief exposure to Acid Tyrode’s solution (pH 2.5), were seeded onto a feeder layer of CF-1 mouse embryonic fibroblast (MEF) cells mitotically inactivated by mitomycin-C treatment (3 h, 10 μg/ml) at passage 3. The MEF medium was replaced with ‘outgrowth medium’ which included DMEM/Nutrient Mixture Ham’s F12 (DMEM/F-12, Gibco) supplemented with 15% fetal calf serum (HyClone), 1 mM sodium pyruvate, 2 mM L-glutamine, 100 units/ml penicillin-streptomycin, 0.1 mM 2-β-mercaptoethanol, 1% non-essential amino acids (NEAA) (all from Gibco), and the factors; 10 ng/ml human recombinant leukemia inhibitory factor (hrLIF; Peprotech) and 10 ng/ml human recombinant basic fibroblast growth factor (hrbFGF; Peprotech). Primary outgrowths began to emerge after approximately 5 days, based on the embryo quality and conditions. Following the emergence of outgrowths, the medium was switched to the outgrowth medium without hrLIF to initiate differentiation. After incubation in the medium for 4–5 days, the medium was subsequently changed to inductive fibroblast medium (IFM; DMEM/F12 medium, supplemented with 10% FBS, the cytokines, 20 ng/ml Activin A, and 10 µg/ml insulin). After 5–7 days of culture in the IFM, the differentiated cell clumps formed within the outgrowths were dissociated with TrypLE Express (Gibco) and plated onto a cell culture dish without MEFs. Each derivative was frozen in FBS based medium supplemented with 8% (v/v) DMSO and recovered with high viability.

### Cell size measurement and growth curves

Countess^®^ Automated Cell Counter and Countess^®^ cell counting chamber slides were purchased from Invitrogen (Carlsbad, CA). A cell count was performed by mixing 20 μl of a sample with 10 μl of 0.8% trypan blue solution, afterwards, the mixture was loaded into the chamber slide to count the cells.

### RNA-seq library preparation and transcriptomic analysis

The RNA from FF and EF cells was extracted using RNeasy Mini Kit (Invitrogen). Libraries for sequencing were prepared using the QIAseq FX Single Cell RNA Library Kit following the manufacturer’s protocol (Qiagen). Triplicate biological replicates were performed for each group. An Illumina Hiseq 4,000 sequencer was used for paired-end sequencing with read length at 150 bp. On average, 25 million reads were obtained for each library. Quality of the RNA-Seq reads was evaluated by FastQC (Babraham Bioinformatics). Low quality reads and adapter contaminations were trimmed using Trimmomatic (Bolger et al., 2014). RNA-seq data were then aligned to the porcine reference genome (Sscrofa11.1) by using STAR (Dobin et al., 2013). The identification of differentially expressed genes (DEGs) was performed by using EdgeR (Robinson et al., 2010). KEGG pathway analysis was performed using clusterProfiler (Wu et al., 2021).

### Gene targeting in EFs

To validate the feasibility of genome editing in EFs, *NANOS3* was chosen for targeting. Candidate high-ranking guide RNAs (sgRNA) were designed using CRISPR design tools (http://chopchop.cbu.uib.no/). The sgRNAs (Integrated DNA Technologies, Coralville, IA) were incubated with SpCas9 Nuclease 3NLS protein (Synthego, Redwood City, CA) at a molar ratio of 1:3 (20 pmol Cas9/60 pmol sgRNA) at room temperature (RT) for 10 min. Single-stranded oligodeoxynucleotides (ssODNs; 100 μM) containing the in-frame 3X-flag tag and translational stop codon were nucleofected alongside Cas9 ribonucleoproteins (RNPs) into 2 × 10^5^ cells using a P3 Primary Cell 4D-Nucleofector X Kit and Nucleofector™ 4D (Lonza, Basel, Switzerland) according to the manufacturer’s recommendation. The experiments were performed using at least three biological replicates (n = 3) and two independent experiments. Following nucleofection, the cells were allowed to recover for 5 days and then plated at a low density onto a 10-cm dish to establish isolated colonies. Ten days later, individual colonies were picked based on morphology and replated into individual wells of 48-well plates. When cells reached near confluency (80%–90%), half of the cells were used for genomic DNA (gDNA) isolation for genotyping analysis, and the other half were cryopreserved. For zygotic injection, the RNPs were pre-complexed by incubating for 10 min at 37°C and diluted to a final concentration of 25 ng/μl for microinjection. Microinjection was performed 4 h after electrical parthenogenetic activation.

### Genotyping analysis

The gDNA from each colony was isolated by using DNA Isolation micro Kit (Norgen) according to the manufacturer’s instructions. PCR was performed by using KOD Hot start mastermix (Novagen) by using the following conditions: denaturation and polymerase activation step of 95°C/3 min, 35 cycles of 95°C/20 s, 60°C/10 s, 70°C/10 s, and the final extension step of 70°C/5 min. The primer sequence and information are provided in Supplementary Table S1. The PCR products were purified using NucleoSpin gel and PCR clean-up kit (Machery Nagel, Bethlehem, PA), and they were subjected to Sanger sequencing (using the forward PCR primer; Table 1). For genotyping analysis in blastocysts and primary outgrowths, each sample was lysed in lysis buffer (20 mM Tris-Cl (pH 8.0), 1% Tween 20, 1% Nonidet P-40 and 100 μg/ml proteinase K) at 56°C for 50 min and boiled at 95°C for 10 min and used as a template for PCR as above. The Sanger trace data was analyzed by using inference of CRISPR edits (ICE, https://ice.synthego.com/) algorithm to measure the extent of mosaicism in individual blastocysts and outgrowths. The PCR amplicons produced for next-generation sequencing (NGS) were purified using a GFX PCR DNA and Gel band purification kit (GE healthcare). NGS library preparations and paired-end (250 bp) sequencing on an Illumina iSeq™ platform was performed by Genewiz, Inc. Amplicon sequencing data were analyzed with CRISPResso v2 with HDR mode.

### Immunocytochemistry

For immunofluorescence staining, cells were fixed with 4% formaldehyde for 5 min at room temperature (RT). After washing once in PBS with 0.1% BSA, cells were permeabilized in PBS with 0.3% Triton X-100 for 15 min and blocked with SuperBlock™ blocking buffer superblock (Thermo Fisher Scientific Inc.) for 30 min prior to antibody incubation. Primary and secondary antibodies were diluted in the blocking solution. Incubation with the primary antibodies was performed at 4°C overnight and secondary antibody at RT for 1 h. The following primary antibodies were used: mouse anti-Vimentin, 1:100 (Santa Cruz sc-6260), rabbit anti-PCNA,1:100 (Abcam ab15580), rabbit, anti-a-SMA, 1:100 (Abcam ab5694). Secondary antibodies were Alexa goat-anti-mouse-647, 1:1,000 (LifeTech A-21121) and Alexa goat-anti-rabbit-488, 1:1,000 (LifeTech A-11034). The nuclei were counterstained with 5 mg/L DAPI (Thermo Fisher Scientific Inc.) for 5 min.

### Statistical analysis

The statistical analysis was performed using GraphPad Prism software. The data was expressed as mean ± SD. For normally distributed data, *t*-test was used for comparison between two independent samples, and one-way analysis of variance (ANOVA) was used for comparisons of multi-samples. A *p*-value of <0.05 was considered as statistically significant. The number of replicates in each experimental setting and statistical significance were shown in each figure legend.

## Results

### Generation of primary fibroblast-like cells from blastocyst outgrowths

**FIGURE 1:**
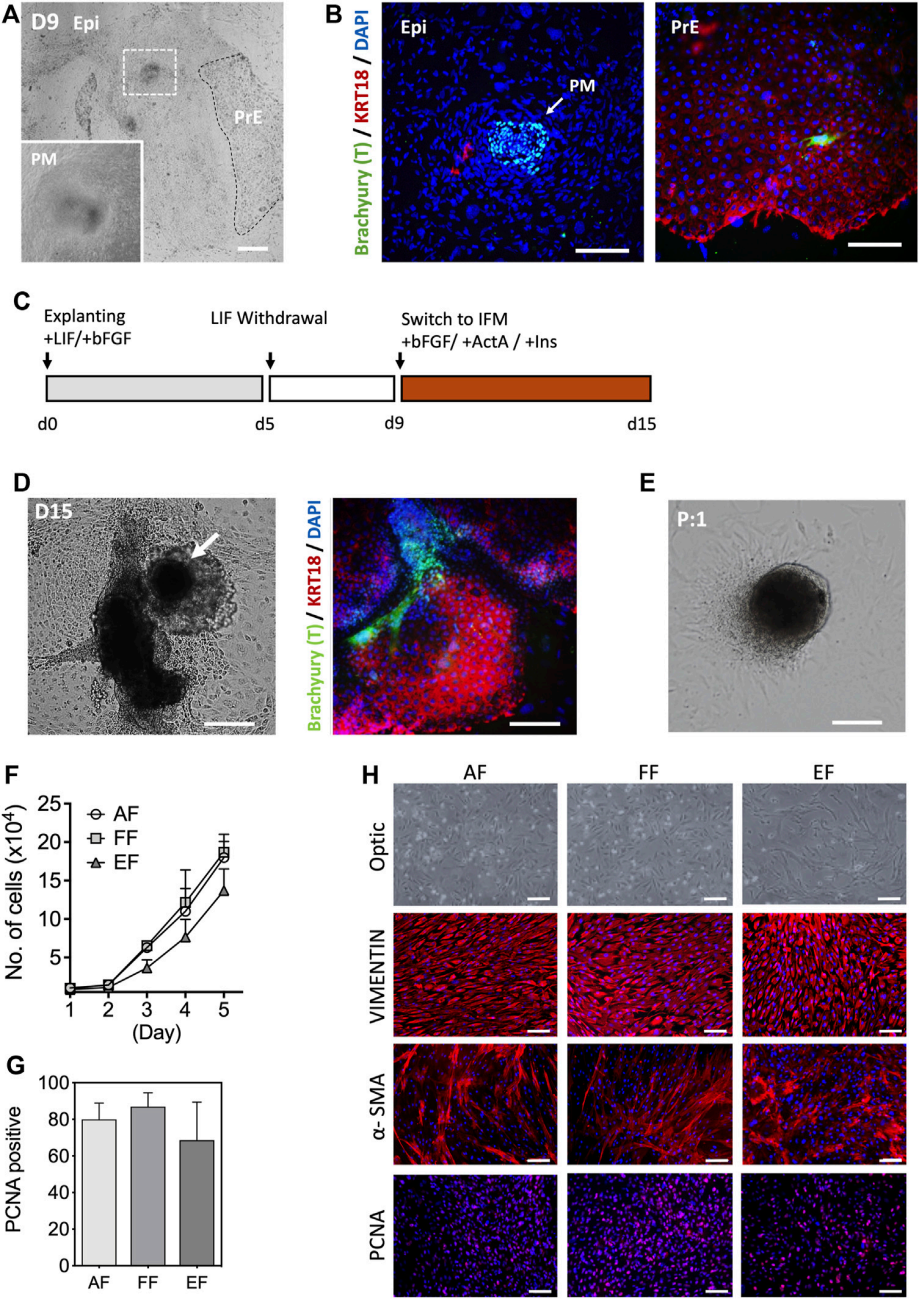
Characterization of embryonic fibroblasts (EF) derived from blastocyst outgrowths. **(A)** Representative images of d9 primary blastocyst outgrowth. Differentiated cell aggregates appear in outgrowths following explanting blastocysts in culture (white dotted, inlet). **(B)** Immunofluorescence staining indicated that the differentiated cell clusters were positive for a mesenchymal marker Brachyury (T; arrow), but negative for epithelial KRT18 after 9 days of differentiation in the hLIF withdrawal medium. **(C)** Schematic of the differentiation protocol. **(D)** Morphological appearance of differentiated cells and immunofluorescence staining of T and KRT18 in d15 primary blastocyst outgrowths. **(E)** The population of spindle-shaped cells grew from the edges of explants following subculture. **(F)** Cell growth curves for the three fibroblast groups: five AFs from a neonatal ear biopsy, five FFs from day 28–50 fetuses, and six EFs from day 7 blastocysts fertilized *in vitro*. Ten thousand cells (passage 3) were used for the experiment (n = 3). **(G)** The mean number of PCNA positive nuclei. In this study, the fetal fibroblast cells displayed higher PCNA positivity than the adult and embryonic fibroblasts, but there were no statistical differences in the number of PCNA positive cells (ANOVA, *p* < 0.05). **(H)** Representative immunostaining images of VIMENTIN, α-SMA, and PCNA marker expression in three fibroblasts. EPI, epiblast, PM, primary mesenchyme, PrE, primitive endoderm. Data is presented as the mean ± SD. Bar, 100 µm.

In the conceptus, primary fibroblasts arise from the primary mesenchyme as the epiblast (Epi) cells undergo an epithelial-to-mesenchymal transition (EMT) during gastrulation (Popovic et al., 2019; LeBleu and Neilson, 2020). Several lines of evidence show that primary fibroblasts can be obtained from the embryonic outgrowths during the embryonic stem cell (ESC) derivation process (D'Angelo et al., 2018). Porcine blastocysts on day 7 of culture that were either hatched or zona denuded were seeded onto mitotically inactivated MEF cultures in the outgrowth medium supplemented with recombinant hbFGF and hLIF. Primary outgrowths begin to emerge after 5 days in culture. Based on a spontaneous differentiation protocol involving LIF withdrawal, we assessed the efficiency of mesenchymal differentiation by analyzing the expression of mesenchymal marker Brachyury (T) and epithelial cytokeratin (KRT)18 in the primary blastocyst outgrowths. Four days after switching to the hLIF withdrawal medium, a tight cluster of T-positive (+) and KRT18-negative (-) cells were identified in the blastocyst outgrowths, which is a key characteristic of mesenchymal cells, while the other extraembryonic cell types (trophoblast and primitive endoderm) show an opposing trend (T- and KRT18+) [Figures 1A,B]. However, the emergence of primary mesenchyme in primary blastocyst outgrowths was observed sporadically and occasionally, so the approach based on spontaneous differentiation was not consistent. In an effort to improve efficiency, d9 blastocyst outgrowths were switched to IFM differentiation medium supplemented with Activin A and insulin from 9 to 15 days, and compared to the outgrowths cultured without supplements [Figure 1C]. Combined induction with Activin A and insulin rendered 33.2% of the outgrowths positive for T, compared to 12.5% without (*p* < 0.05, n = 5) supplementation, indicating that IFM containing Activin A and Insulin promote primary mesenchyme (T+) development after continued culture for 2 weeks in the medium without passaging [Figure 1D]. Following the first passage, the tight clusters of cells remained intact, from which fibroblast-like cells grew out and serially propagated in culture in a manner similar to the cells from fetal tissues [Figure 1E]. The growth rate of the blastocyst-derived embryonic fibroblasts (EF) cells was comparable to that of fibroblasts from adult (AF) and fetal (FF) origins, although EFs had a slightly lower growth rate (not significant) than AF and FF cells [Figure 1F]. As shown in Figures 1G,H, the EFs displayed typical spindle-shaped morphology and expression of the fibroblast molecular markers, vimentin (VIM) and α-smooth muscle actin (α-SMA), and the proliferating cell nuclear antigen (PCNA). The expression of α-SMA was lower in AFs and FFs than EFs, whereas VIM was consistently expressed in fibroblast cells regardless of origins. In particular, the early-passage EFs were a mixed population (e.g., shape, cell size, growth rate, or marker expression) of large and small cells with varied expression of α-SMA and PCNA [Supplementary Figures S1A,B]. As illustrated in Figure 1C, our results suggest that blastocyst outgrowths could differentiate into cells with characteristics typical of fibroblast phenotype.

### Comparison of global expression profiles of fibroblasts from different origins

**FIGURE 2:**
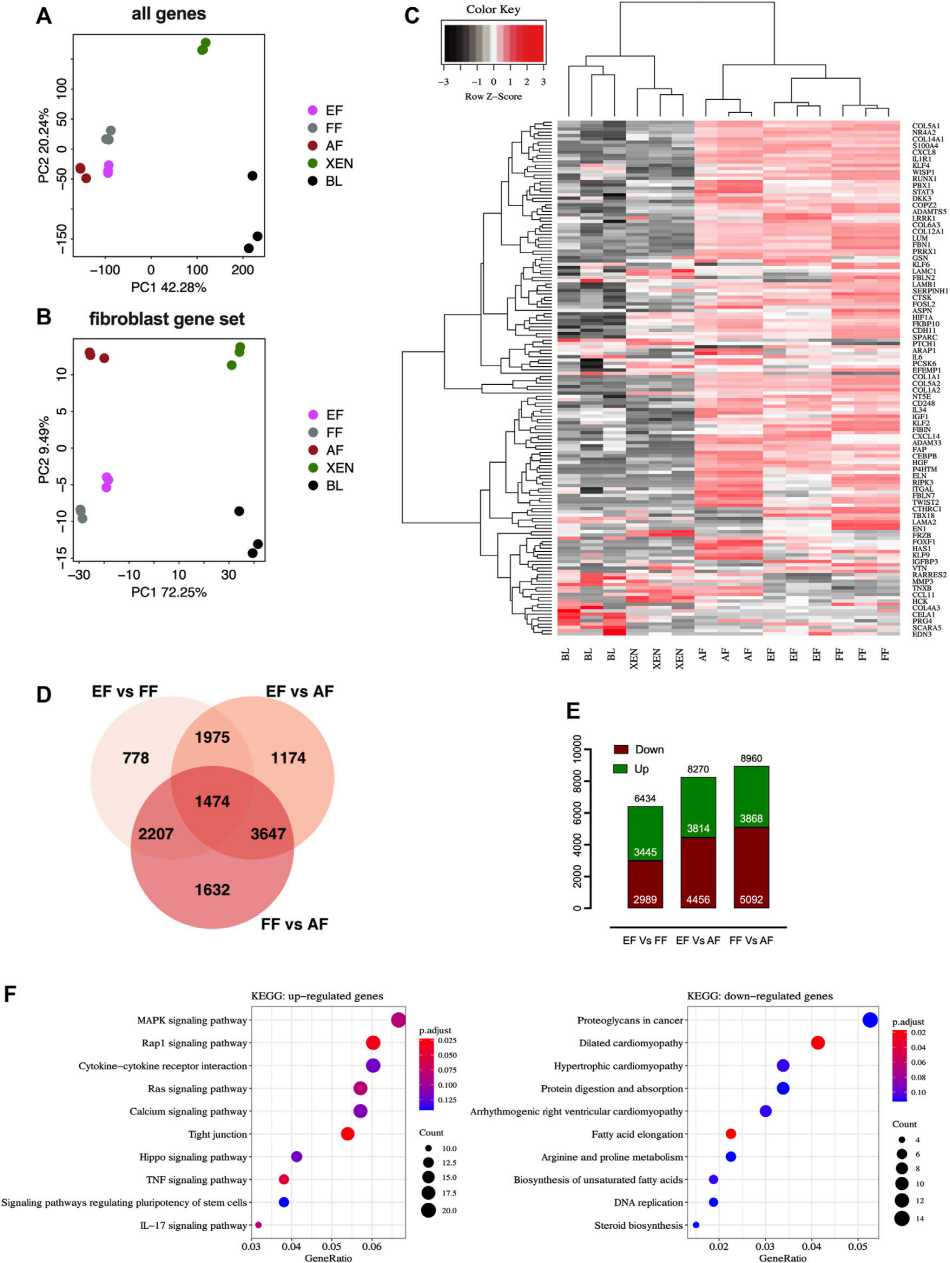
RNAseq analysis of fibroblasts from different origins. Principal component analysis (PCA) plots of RNA-seq data based on **(A)** global gene expression or a candidate list of **(B)** fibroblast marker genes. The two axes PC1 and PC2 represent the first two principal components identified by the analysis. Each color code denotes different cell types in the scheme in the middle of the plots. **(C)** Gene expression heatmap of 156 genes in the fibroblast gene-set. **(D)** Venn diagram showing that numbers in each segment represent differentially expressed genes (DEGs) that are not shared, and in overlapping segments-the shared DEGs among the fibroblasts. **(E)** Bar plot of the number of differentially expressed genes (DEGs). Downregulated genes are depicted in red, and upregulated genes are depicted in green. **(F)** KEGG pathway enrichment analysis of DEGs. Bubble plots showing the significant pathways for up-and downregulated DEGs, in which the −log10 of the adjusted *p*-value are represented by colors. The number of DEGs in the pathway is indicated by the circle area, and the circle color represents the range of the adjusted q values (FDR). We display the top 10 pathway terms enriched by KEGG database. AF, adult fibroblast; EF, embryonic fibroblast; FF, fetal fibroblast; XEN, extraembryonic endodermal stem cells; BL, blastocyst.

We performed comparative RNA-seq transcriptomic analysis of EF cells. Three cell lines from each group, EFs from *in vitro* d7 blastocysts, and FFs from d28 fetuses were analyzed alongside publicly available datasets for AFs (NCBI GSE146494), XEN cells (NCBI GSE128149), Blastocysts (BL; NCBI GSE128149). The principal component analysis (PCA) plots based on the global expression profile clustered the fibroblasts of embryonic, fetal, or adult origin together confirming fibroblastic profile of EF cells [Figure 2A]. The PCA plot based on the candidate fibroblast-marker gene-set [Supplementary Table S2] showed that EFs and FFs were grouped closely compared to AFs [Figure 2B]. Hierarchical clustering using fibroblast marker genes (156) also showed a pattern consistent with principal clustering analysis results [Figure 2C]. Venn diagram comparing differentially expressed genes (DEGs) revealed that AFs transcriptome is different from EFs and FFs, with 8,270 DEGs compared to EFs and 8,960 DEGs compared to FFs, whereas there are 6,434 DEGs between EFs and FFs [Figure 2D]. The smaller number of up-and down-regulated genes in the EFs *versus* FFs indicated that they are more comparable to each other than to AFs [Figure 2E]. Functionally, as revealed by KEGG-pathway analysis of the clusters (EF + FF vs. AF), many genes down-regulated in AFs were related to ribosome biogenesis, RNA degradation, protein digestion, and absorption/ECM-receptor interaction, suggesting down-regulation specifically of post-transcriptional regulation [Supplementary Figures S2A,B]. Furthermore, the enrichment analysis of EFs vs. FFs showed that the DEGs in EFs were primarily enriched for fibroblast growth factor (FGF) signaling including MAPK, Ras, Rap1, and TNF kinase pathways [Figure 2F]. This result indicates that growth factor and cytokines (bFGF and LIF) used for the cell derivation may lead to constitutive activation of relevant pathways that in turn changes gene expression patterns. With regard to the biological relevance of these findings, the observed changes in the presence of signaling molecules, and their importance for phenotypic characteristics remain to be seen.

### *In vivo* developmental potential of cloned embryos generated from embryonic fibroblasts

**FIGURE 3:**
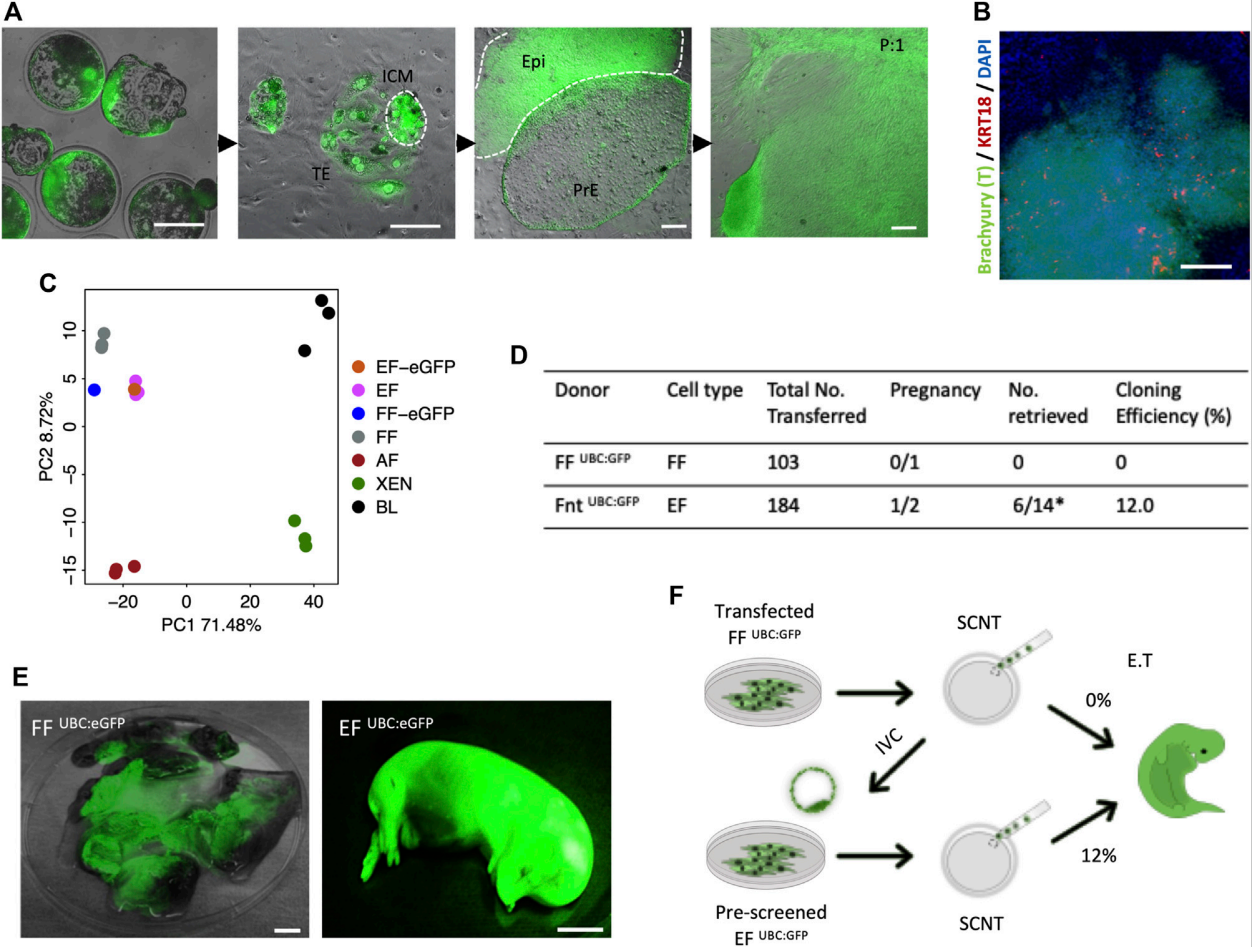
*In vivo* development of 40-day fetuses following SCNT. **(A)** Representative images of the blastocyst outgrowths and the derivation of EF and PRE cells. The constitutive hUBC promoter-driven GFP transgenic FF (FF ^UBC:eGFP^) were used for generating SCNT. The establishment of primary outgrowths from SCNT embryonic explants on day 2 of culture and the emergence of PrE and Epi in d9 primary outgrowths. After the first passage, fibroblast-like cells grew from the differentiated cell clump (EF ^UBC:eGFP^). **(B)** Immunofluorescence staining confirmed that the differentiated cell clusters were Brachyury positive but KRT18 negative after 15 days of differentiation in the IFM medium. **(C)** PCA plot of FF ^UBC:eGFP^ and EF ^UBC:eGFP^. **(D)** Pregnancy and cloning efficiency rate of pig embryos generated from FF ^UBC:eGFP^ and EF ^UBC:eGFP^ both on the same genetic background. **(E)** Representative images of mummified fetuses from FF ^UBC:eGFP^ (left) and normal developmentally competent fetuses from EF ^UBC:eGFP^ cloned embryos (right) expressing ubiquitous eGFP on day 40 of pregnancy. **(F)** Schematic summary of the genome-edited experiments highlighting improved SCNT efficiencies with the use of EF as nuclear donor cells in SCNT. Bar, 1 mm.

As has been proven previously, the derivation of XEN cells from genetically modified porcine FFs deemed ‘unfit’ for SCNT, and subsequent generation of cloned animals from the newly established XEN cells with enhanced cloning efficiencies, highlighted novel opportunities to generate cloned animals from desirable, yet cells not clonable by SCNT (Park et al., 2021). To determine whether a similar effect on SCNT could be achieved with EFs, we have generated the cloned blastocysts from previously reported transgenic fibroblast cells FF ^UBC:GFP^ [labeled “UBC:eGFP”; Figure 3A]. Consistent with the process for derivation of EFs from *in vivo* or *in vitro* fertilized embryos, outgrowths from the cloned embryos in culture with a tight cluster of mesenchymal-like cells (Brachyury +/KRT18 -) were observed by day 9 [Figure 3B], and following the first subculture, large cell clusters migrated out onto the plate surface [Figure 3A]. From individual embryonic outgrowths, we induced EF and XEN cells, and the transcriptomic analysis was performed on the established EF cells. As expected, the PCA plot confirmed that the EFs clustered closely with FFs and that the EFs closely resemble FFs [Figure 3C]. To examine the *in vitro* developmental competence of EFs, we performed SCNT using EF ^UBC:eGFP^ and FF ^UBC:eGFP^, showing that there were no significant differences in the cleavage (74%) and blastocyst formation percentages (36%) compared to the percentages with FFs used for generating the UBC:GFP reporter transgenic cells (Data not shown). A total of 184 reconstituted embryos from EF ^UBC:GFP^ were transferred into two surrogate gilts, and one pregnancy was established, while 103 cloned from FF ^UBC:GFP^ failed to establish a pregnancy. Of the 14 cloned fetuses retrieved at day 40 of pregnancy, six fetuses developed normally and their size is compatible with their embryonic stage, while the remainder were developmentally delayed (8/14) [Figure 3D]. All of them exhibited a strong GFP expression [Figure 3E]. Taken together, these results confirm that the EFs could potentially improve SCNT efficiencies and are in line with the improved SCNT efficiencies observed with the use of rederived embryonic cells as nuclear donors [previously observed (Park et al., 2021)] [Figure 3F].

### Embryonic fibroblasts as a source for gene targeting

**FIGURE 4:**
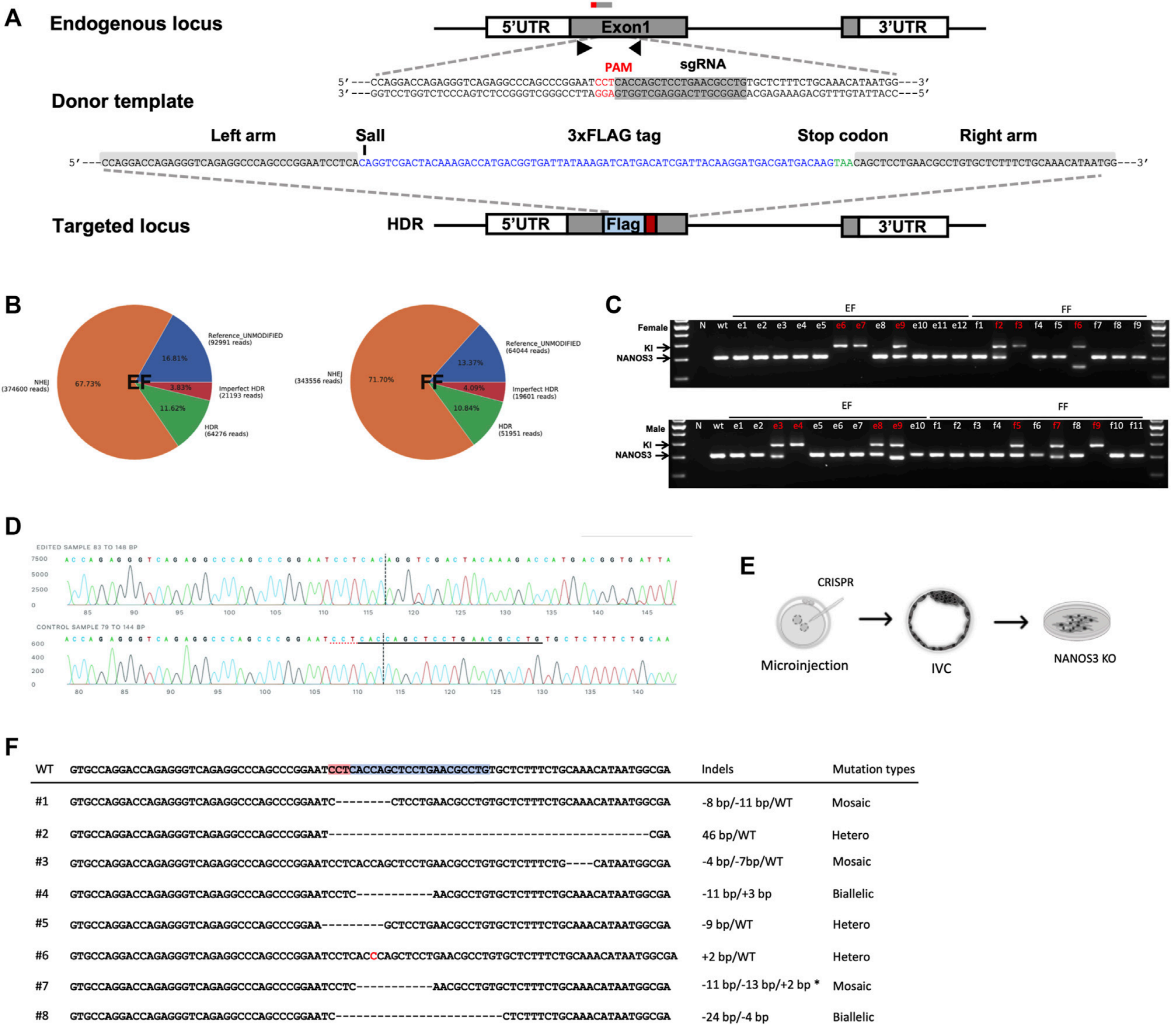
CRISPR/Cas9-mediated modification of *NANOS3* in embryonic fibroblasts. **(A)** Strategy for disruption of *NANOS3* with insertion of a premature translational-stop codon (PTC). A candidate ssODN was designed containing a 3xFLAG epitope tag and a PTC (TAA) flanked by 40 nt homology arms corresponding to the region to be edited. The cassette contains restriction enzyme cut sites (SalI) to assist in RFLP-based assay. **(B)** Quantification and visualization of the outcomes of genome editing. Pie charts summarizing the frequencies of events in the *NANOS3* loci from targeted amplicon sequencing in EF and FF cells. When an expected HDR amplicon is provided, CRISPResso classifies sequence reads as blue, unmodified alleles; orange, NHEJ mutations; red, imperfect HDR; green, HDR alleles as distinct outcomes. **(C)** Genomic sequencing showing homozygous/heterozygous mutant DNA carrying the intended edit. Using the primers indicated by the arrows, the amplicons from genomic DNA were resolved on a gel revealing HDR (374 bp) and wild-type (Wt, 300bp) and/or NHEJ (<300bp) fragments. **(D)** Sanger sequencing of clonal EFs showing precise gene knockin at the target site. **(E)** Schematic outline for the direct generation of knockout *NANOS3* ^−/−^ embryonic fibroblasts from outgrowths. **(F)** Sanger sequencing of established EFs *via* zygotic injection shows a range of mutations at the target site.

Based on the observation that EFs constitute an improved cell source for SCNT, and to harness the true potential of EFs as a tool for generating genetically modified offspring, we tested two distinct approaches: 1) targeted modification directly in the EFs, and 2) generating EFs from genome edited embryos. As a candidate for genetic manipulation, we chose *NANOS3* to test the feasibility of this approach. The candidate gene- *NANOS3* belongs to the NANOS family of proteins, which have an essential role in germ cell development. Mutations in *NANOS2* cause germ cell loss in male pigs but not in females, as reported in our prior publication (Park et al., 2017). *NANOS3* was shown to have an essential role in both male and female germ cells throughout spermatogenesis and oogenesis (Tsuda et al., 2003; Ideta et al., 2016). First, we transiently transfected cells with a control plasmid (pmaxGFP) to assess the feasibility and patency of transfection of nucleic acids into EFs. Fluorescence-activated cell sorting (FACS) analysis showed that the EFs are compatible and tolerate nucleofection, with 72.0% of EFs showing GFP expression compared to 83.4% for FFs [Supplementary Figures S4A,B]. Next, to evaluate the delivery and efficient activity of SpCas9 at the target site, we designed two candidate sgRNAs targeting *NANOS3*, precomplexed with SpCa9 protein, and the resulting ribonucleoprotein complexes (RNPs) nucleofected into EFs. Following a period of recovery (2–3 days) the efficiencies of targeting were assessed by targeted amplicon sequencing (Illumina). We identified successful delivery and high efficiency of on-site editing with candidate guide#1 (indel rate; 82.32% for guide1 and 47.62% for guide 2). The high activity, Guide#1 was selected for subsequent experiments [Supplementary Figure S4C]. To evaluate CRISPR-mediated, homology-directed repair (HDR) based knock-in at the target site, ssODNs containing an in-frame three Flag epitopes in tandem (3xFlag), and a translational stop codon to prematurely truncate the protein before its zinc finger domain consisting of two consecutive C2HC-type zinc finger motifs, and a novel SalI restriction site for a restriction fragment polymorphism (RFLP) assay was designed for HDR mediated knock-in into and subsequent knockout of the *NANOS3* allele [Figure 4A]. We delivered RNPs alongside ssODNs into candidate female EF and FF cells. Following transfection, and screening *via* RFLP and Sanger sequencing, we identified 11.6% of HDR and 67.7% of non-homologous end joining (NHEJ) events within the EFs; and 10.8% HDR and 71.7% NHEJ events within the FFs, respectively. The overall editing rates in EFs were similar to that of FFs (n = 3, *p* < 0.01) [Figure 4B and Supplementary Figure S4D]. Following confirmation of editing, the transfected EFs were plated at a low density to establish clonal lines. Among the established lines, a total of 3 colonies (2 homozygous and 1 biallelic) from female and 4 colonies (1 homozygous and 3 biallelic) male EFs were identified to have precise HDR targeting events as confirmed by RFLP and Sanger sequencing [Figures 4C,D]. Furthermore, a similar strategy was employed with female and male FFs. The results showed that 3 colonies (1 homozygous and 2 biallelic) from female and 3 colonies (1 homozygous and 2 biallelic) from male FFs were precisely corrected [Supplementary Figure S4E]. These colonies were cryopreserved for future studies.

Secondly, we investigated the feasibility of gene targeting directly in the embryos (zygotic injection) and establishing clonal EFs from the edited embryos [Figure 4E]. As above, the guide#1 which exhibited a high editing efficiency was used, and the RNPs targeting the *NANOS3* were injected into parthenogenetic zygotes (n = 65), a total of 58 embryos developed to the blastocyst stage and 20 blastocysts were utilized to screen for mutation frequencies. Most of the screened embryos (17/20) displayed on-site activity and displayed a range of mutations, including either sgRNA site–spanning insertions/deletions or deletion distantly from the sgRNA target site, with a high mosaicism frequency (11 of 20 were mosaic) [Supplementary Figure S4F]. Following confirmation of editing, the remaining expanded d7 blastocysts (n = 38) were used for EF derivation through blastocyst outgrowth. We obtained a total of 8 EF lines from these outgrowths, with 1 candidate line showing biallelic mutations [Figure 4F]. Taken together, we showed the feasibility of establishing edited EF lines, with implications for generating gene-edited animals.

## Discussion

The recent development of GE technologies, principally the CRISPR/Cas system has now afforded facile genetic modification in pigs and other livestock species. The GE piglets can be generated by the delivery of CRISPR reagents into somatic cells, typically FF followed by SCNT. Although infrequent, there is a concern of reduced cell viability in clonally derived modified cells following transfection, which can have an adverse impact on SCNT efficiency (Keefer, 2015; Matoba and Zhang, 2018; Gouveia et al., 2020). While GE methods have significantly improved in recent years, the selection of prospective donor cells suitable for SCNT among the potential multitude of clonal lines with the correct targeting events remains difficult. Our previous study demonstrated that embryo-derived primary cells can serve as an alternative source of donor cells with an enhanced efficiency (Park et al., 2021). However, there is a drawback due to the intrinsic properties of such cells, including the relatively higher costs for derivation, and difficulty in isolating clonal populations for GE. Relying on fibroblasts directly obtained from blastocyst outgrowths is highly advantageous for the following reasons: 1) in contrast to culturing stem cells, fibroblasts are easily cultured and maintained *in vitro*, allowing their use as their equivalents from fetuses; 2) preselect donor cells carrying the targeted modifications for a successful outcome following SCNT; and 3) serial editing followed by SCNT and stacking of multiple genetic engineering events, bypassing the need to establish FF by embryo transfer between each targeting round.

The primary mesenchyme is detectable in spontaneously differentiating blastocyst outgrowths and pluripotent stem cells (D'Angelo et al., 2018). Based on the conditions shown to differentiate ESC to fibroblasts *in vitro* (Wang et al., 2014; Bao et al., 2016), we have also observed that the reported differentiation conditions including a combination of bFGF, Activin A, and Insulin have resulted in similar differentiation to primary fibroblasts from primary embryonic outgrowths. By comparing transcriptome signatures of fibroblasts that were established from different developmental stages, the newly established EFs exhibited marked similarities to FFs as compared to AFs, reflecting the developmental proximity to the fetal developmental stage, even though the cultured fibroblasts from different origins had similarities in morphology, marker expression, and proliferation. Our efforts to derive the EF cells have been met with reliable but modest success (33.2%) due to high heterogeneity among the established embryos and the resulting embryonic outgrowths. Further optimization is needed to directly differentiate the outgrowths into specific cell types including fibroblasts. The proposed approach in this report is expected to serve as a baseline for improving our understanding of the developmental signaling mechanisms that are crucial for specifying EF cell formation and help fuel further technical refinements and validation efforts.

Culturing of the embryos beyond the blastocyst stage, assessing outgrowth development, and an *in vitro* implantation assay are used as useful experimental models to investigate the predictive capacity of embryo quality for successful implantation following embryo transfer (Popovic et al., 2019). Although genome modification with precise modification *via* HDR for introducing point mutations (or knocking-in transgenes) is desirable over the error-prone NHEJ, it is often associated with an increase in cytotoxicity that could negatively impact the generation of GE animals using SCNT. In this context, pre-selection of developmental competence of the edited embryos, and their subsequent ability to generate explants and allow clonal selection of EFs may serve as a positive predictor of their eventual developmental competence and a high potential for successful implantation and pregnancy. As shown in this report, rederivation of a genetically modified EF cell line has resulted in high cloning efficiency (12%). Second trimester (day 40) was chosen as an endpoint because, 1) healthy fetuses at this stage (six in this study) are relatively a good predictor of a successful pregnancy outcome, and 2) the fetuses at this stage do not initiate breathing and will not need to be euthanized. Going forward, these results will need to be validated further by allowing the pregnancies progress to term and the birth of live offspring.

Extensive reports on the high rates of generating genotypic mosaicism induced in CRISPR/Cas9 mediated gene-edited animals generated by zygote injection have already been published (Park et al., 2016; Zhou et al., 2016; Burkard et al., 2017; Hennig et al., 2022). From a technical perspective, previous reports of generating GE animals by zygotic injections among various species with the goal of reducing mosaicism have met with limited success (Hashimoto et al., 2016; Ma et al., 2017; Vilarino et al., 2017; Li et al., 2020). Despite the high targeting efficiency in the zygotes and relative simplicity of the procedure, a great deal of work remains to be performed on this issue of mosaicism and inheritance of intact alleles, confounding the phenotype of resulting mutations and alleles among the G0 founders. Taking the variable editing outcomes into consideration from zygotic or cellular engineering, and the low predictability of edited cells in generating robust embryos *via* SCNT for a successful pregnancy, the proposed method in this report can be leveraged for rapid and efficient generation of GE donor EFs from *in vitro* cultured embryos followed by zygotic injection for efficient generation of GE cloned pigs. Overall, the proposed methodology is expected to decrease time and costs, bypass the need for surgical procedures, animal suffering, and sacrificing the surrogates for establishing FFs, culminating in the overall improved efficiency of the process for generating genetically modified pigs, and other livestock animals.

## Conclusion

This study demonstrated that blastocyst outgrowths can differentiate directly into primary EFs, which are similar to fetal derived fibroblasts in morphology, marker expression, and transcriptomic signature, but further studies are warranted to further standardize the fibroblast derivation protocol from blastocyst outgrowths. Importantly, EFs provide the ability to isolate developmentally competent SCNT donor cells from genome edited zygotes, and also as a source for somatic genome-editing, potentially facilitating serial and limitless genome editing capability without a loss of proliferative ability.

## Data Availability

The datasets presented in this study can be found in online repositories. The names of the repository/repositories and accession number(s) can be found below: SRA database accession number: PRJNA885580.

## References

[B1] Bao X. Lian X. Hacker T. A. Schmuck E. G. Qian T. Bhute V. J. (2016). Long-term self-renewing human epicardial cells generated from pluripotent stem cells under defined xeno-free conditions. Nat. Biomed. Eng. 1, 0003. 10.1038/s41551-016-0003 28462012 PMC5408455

[B2] Bolger A. M. Lohse M. Usadel B. (2014). Trimmomatic: A flexible trimmer for Illumina sequence data. Bioinformatics 30, 2114–2120. 10.1093/bioinformatics/btu170 24695404 PMC4103590

[B3] Burkard C. Lillico S. G. Reid E. Jackson B. Mileham A. J. Ait-Ali T. (2017). Precision engineering for PRRSV resistance in pigs: Macrophages from genome edited pigs lacking CD163 SRCR5 domain are fully resistant to both PRRSV genotypes while maintaining biological function. PLoS Pathog. 13, e1006206. 10.1371/journal.ppat.1006206 28231264 PMC5322883

[B4] Campbell K. H. Mcwhir J. Ritchie W. A. Wilmut I. (1996). Sheep cloned by nuclear transfer from a cultured cell line. Nature 380, 64–66. 10.1038/380064a0 8598906

[B5] D'angelo W. Chen B. Gurung C. Guo Y. L. (2018). Characterization of embryonic stem cell-differentiated fibroblasts as mesenchymal stem cells with robust expansion capacity and attenuated innate immunity. Stem Cell Res. Ther. 9, 278. 10.1186/s13287-018-1033-8 30359317 PMC6203291

[B6] Dobin A. Davis C. A. Schlesinger F. Drenkow J. Zaleski C. Jha S. (2013). Star: Ultrafast universal RNA-seq aligner. Bioinformatics 29, 15–21. 10.1093/bioinformatics/bts635 23104886 PMC3530905

[B7] Gouveia C. Huyser C. Egli D. Pepper M. S. (2020). Lessons learned from somatic cell nuclear transfer. Int. J. Mol. Sci. 21, E2314. 10.3390/ijms21072314 PMC7177533 32230814

[B8] Hashimoto M. Yamashita Y. Takemoto T. (2016). Electroporation of Cas9 protein/sgRNA into early pronuclear zygotes generates non-mosaic mutants in the mouse. Dev. Biol. 418, 1–9. 10.1016/j.ydbio.2016.07.017 27474397

[B9] Hennig S. L. Owen J. R. Lin J. C. Mcnabb B. R. Van Eenennaam A. L. Murray J. D. (2022). LincRNA#1 knockout alone does not affect polled phenotype in cattle heterozygous for the celtic POLLED allele. Sci. Rep. 12, 7627. 10.1038/s41598-022-11669-9 35538091 PMC9090918

[B10] Ideta A. Yamashita S. Seki-Soma M. Yamaguchi R. Chiba S. Komaki H. (2016). Generation of exogenous germ cells in the ovaries of sterile NANOS3-null beef cattle. Sci. Rep. 6, 24983. 10.1038/srep24983 27117862 PMC4846992

[B11] Inoue K. Ogonuki N. Mochida K. Yamamoto Y. Takano K. Kohda T. (2003). Effects of donor cell type and genotype on the efficiency of mouse somatic cell cloning. Biol. Reprod. 69, 1394–1400. 10.1095/biolreprod.103.017731 12801984

[B12] Keefer C. L. (2015). Artificial cloning of domestic animals. Proc. Natl. Acad. Sci. U. S. A. 112, 8874–8878. 10.1073/pnas.1501718112 26195770 PMC4517265

[B13] Lebleu V. S. Neilson E. G. (2020). Origin and functional heterogeneity of fibroblasts. FASEB J. 34, 3519–3536. 10.1096/fj.201903188R 32037627

[B14] Li Y. Weng Y. Bai D. Jia Y. Liu Y. Zhang Y. (2020). Precise allele-specific genome editing by spatiotemporal control of CRISPR-Cas9 via pronuclear transplantation. Nat. Commun. 11, 4593. 10.1038/s41467-020-18391-y 32929070 PMC7490392

[B15] Ma H. Marti-Gutierrez N. Park S. W. Wu J. Lee Y. Suzuki K. (2017). Correction of a pathogenic gene mutation in human embryos. Nature 548, 413–419. 10.1038/nature23305 28783728

[B16] Matoba S. Zhang Y. (2018). Somatic cell nuclear transfer reprogramming: Mechanisms and applications. Cell Stem Cell 23, 471–485. 10.1016/j.stem.2018.06.018 30033121 PMC6173619

[B17] Park C. H. Jeoung Y. H. Uh K. J. Park K. E. Bridge J. Powell A. (2021). Extraembryonic endoderm (XEN) cells capable of contributing to embryonic chimeras established from pig embryos. Stem Cell Rep. 16, 212–223. 10.1016/j.stemcr.2020.11.011 PMC7897585 33338433

[B18] Park K. E. Kaucher A. V. Powell A. Waqas M. S. Sandmaier S. E. Oatley M. J. (2017). Generation of germline ablated male pigs by CRISPR/Cas9 editing of the NANOS2 gene. Sci. Rep. 7, 40176. 10.1038/srep40176 28071690 PMC5223215

[B19] Park K. E. Park C. H. Powell A. Martin J. Donovan D. M. Telugu B. P. (2016). Targeted gene knockin in porcine somatic cells using CRISPR/Cas ribonucleoproteins. Int. J. Mol. Sci. 17, E810. 10.3390/ijms17060810 PMC4926344 27240344

[B20] Perisse I. V. Fan Z. Singina G. N. White K. L. Polejaeva I. A. (2020). Improvements in gene editing technology boost its applications in livestock. Front. Genet. 11, 614688. 10.3389/fgene.2020.614688 33603767 PMC7885404

[B21] Popovic M. Bialecka M. Gomes Fernandes M. Taelman J. Van Der Jeught M. De Sutter P. (2019). Human blastocyst outgrowths recapitulate primordial germ cell specification events. Mol. Hum. Reprod. 25, 519–526. 10.1093/molehr/gaz035 31211841 PMC6802404

[B22] Robinson M. D. Mccarthy D. J. Smyth G. K. (2010). edgeR: a Bioconductor package for differential expression analysis of digital gene expression data. Bioinformatics 26, 139–140. 10.1093/bioinformatics/btp616 19910308 PMC2796818

[B23] Rubinstein C. D. Mclean D. T. Lehman B. P. Meudt J. J. Schomberg D. T. Krentz K. J. (2021). Assessment of mosaicism and detection of cryptic alleles in CRISPR/Cas9-Engineered neurofibromatosis type 1 and TP53 mutant porcine models reveals overlooked challenges in precision modeling of human diseases. Front. Genet. 12, 721045. 10.3389/fgene.2021.721045 34630515 PMC8495252

[B24] Tanihara F. Hirata M. Nguyen N. T. Le Q. A. Hirano T. Otoi T. (2019). Effects of concentration of CRISPR/Cas9 components on genetic mosaicism in cytoplasmic microinjected porcine embryos. J. Reprod. Dev. 65, 209–214. 10.1262/jrd.2018-116 30726783 PMC6584178

[B25] Torres J. Prieto J. Durupt F. C. Broad S. Watt F. M. (2012). Efficient differentiation of embryonic stem cells into mesodermal precursors by BMP, retinoic acid and Notch signalling. PLoS One 7, e36405. 10.1371/journal.pone.0036405 22558462 PMC3340340

[B26] Tsuda M. Sasaoka Y. Kiso M. Abe K. Haraguchi S. Kobayashi S. (2003). Conserved role of nanos proteins in germ cell development. Science 301, 1239–1241. 10.1126/science.1085222 12947200

[B27] Vilarino M. Rashid S. T. Suchy F. P. Mcnabb B. R. Van Der Meulen T. Fine E. J. (2017). Author correction: CRISPR/Cas9 microinjection in oocytes disables pancreas development in sheep. Sci. Rep. 7, 7500. 10.1038/s41598-020-64443-0 32371904 PMC7200707

[B28] Wang R. Wang J. Acharya D. Paul A. M. Bai F. Huang F. (2014). Antiviral responses in mouse embryonic stem cells: Differential development of cellular mechanisms in type I interferon production and response. J. Biol. Chem. 289, 25186–25198. 10.1074/jbc.M113.537746 24966329 PMC4155682

[B29] Wang X. Qu J. Li J. He H. Liu Z. Huan Y. (2020). Epigenetic reprogramming during somatic cell nuclear transfer: Recent progress and future directions. Front. Genet. 11, 205. 10.3389/fgene.2020.00205 32256519 PMC7093498

[B30] Whitelaw C. B. Sheets T. P. Lillico S. G. Telugu B. P. (2016). Engineering large animal models of human disease. J. Pathol. 238, 247–256. 10.1002/path.4648 26414877 PMC4737318

[B31] Whitworth K. M. Green J. A. Redel B. K. Geisert R. D. Lee K. Telugu B. P. (2022). Improvements in pig agriculture through gene editing. CABI Agric. Biosci. 3, 41. 10.1186/s43170-022-00111-9 35755158 PMC9209828

[B32] Wu T. Hu E. Xu S. Chen M. Guo P. Dai Z. (2021). clusterProfiler 4.0: A universal enrichment tool for interpreting omics data. Innovation. 2, 100141. 10.1016/j.xinn.2021.100141 34557778 PMC8454663

[B33] Zhou X. Wang L. Du Y. Xie F. Li L. Liu Y. (2016). Efficient generation of gene-modified pigs harboring precise orthologous human mutation via CRISPR/Cas9-Induced homology-directed repair in zygotes. Hum. Mutat. 37, 110–118. 10.1002/humu.22913 26442986

