# Supplementary material for: Establishment, characterization, and validation of novel porcine embryonic fibroblasts as a potential source for genetic modification
Source: Front Cell Dev Biol. 2022 Nov 10;10:1059710. doi: 10.3389/fcell.2022.1059710 (PMC9685398; doi:10.3389/fcell.2022.1059710)
Supplement: Supplementary file 1 [file DataSheet1.PDF]

Supplemental data

Establishment, characterization, and validation of novel porcine embryonic fibroblasts as a potential source for genetic modification

Chi-Hun Park[1,2], Young-Hee Jeoung[1,2], Luhui Zhang[1], Sai Goutham Reddy Yeddula[1], Ki-Eun Park[2], Jerel Waters[2] and Bhanu P. Telugu[1,2] *

[1] Division of Animal Sciences, University of Missouri, Columbia MO 65211;

[2] RenOVAte Biosciences Inc, Reisterstown, MD 21136

The PDF file includes:

Figure. S1. Characterization of EFs derived from blastocyst outgrowths
Figure. S2. KEGG pathway enrichment analysis of DEGs.
Figure. S3. CRISPR/Cas9-mediated modification of NANOS3 in embryonic fibroblasts
Table. S1. Primers and Oligonucleotides used
Table. S2. Fibroblast gene-set

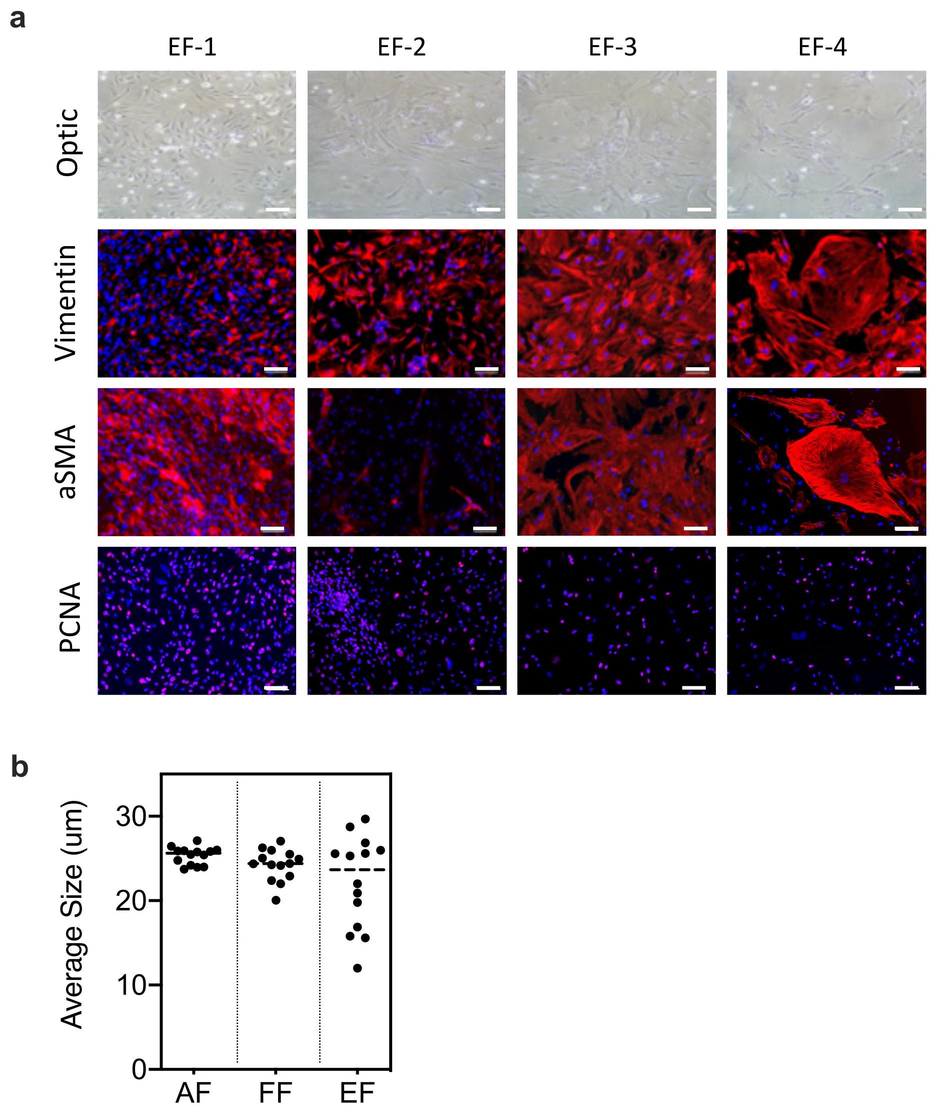

Figure. S1 related to Fig.1 Characterization of EFs derived from blastocyst outgrowths. (a) Representative immunostaining images of VIMENTIN and α-SMA marker expression in EFs. (b) Average cell size was measured using the Countess automated cell counter (n=3). Bar, 100 um.

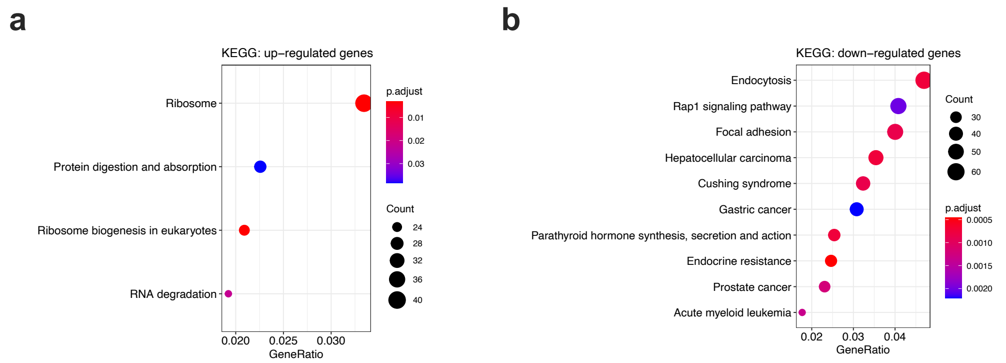

Figure. S2. 2 related to Fig.2 KEGG pathway enrichment analysis of DEGs. Bubble plots showing the significant pathways for up-and downregulated DEGs, in which the −log10 of the adjusted p-value are represented by colors. The number of DEGs in the pathway is indicated by the circle area, and the circle color represents the range of the adjusted q values (FDR).

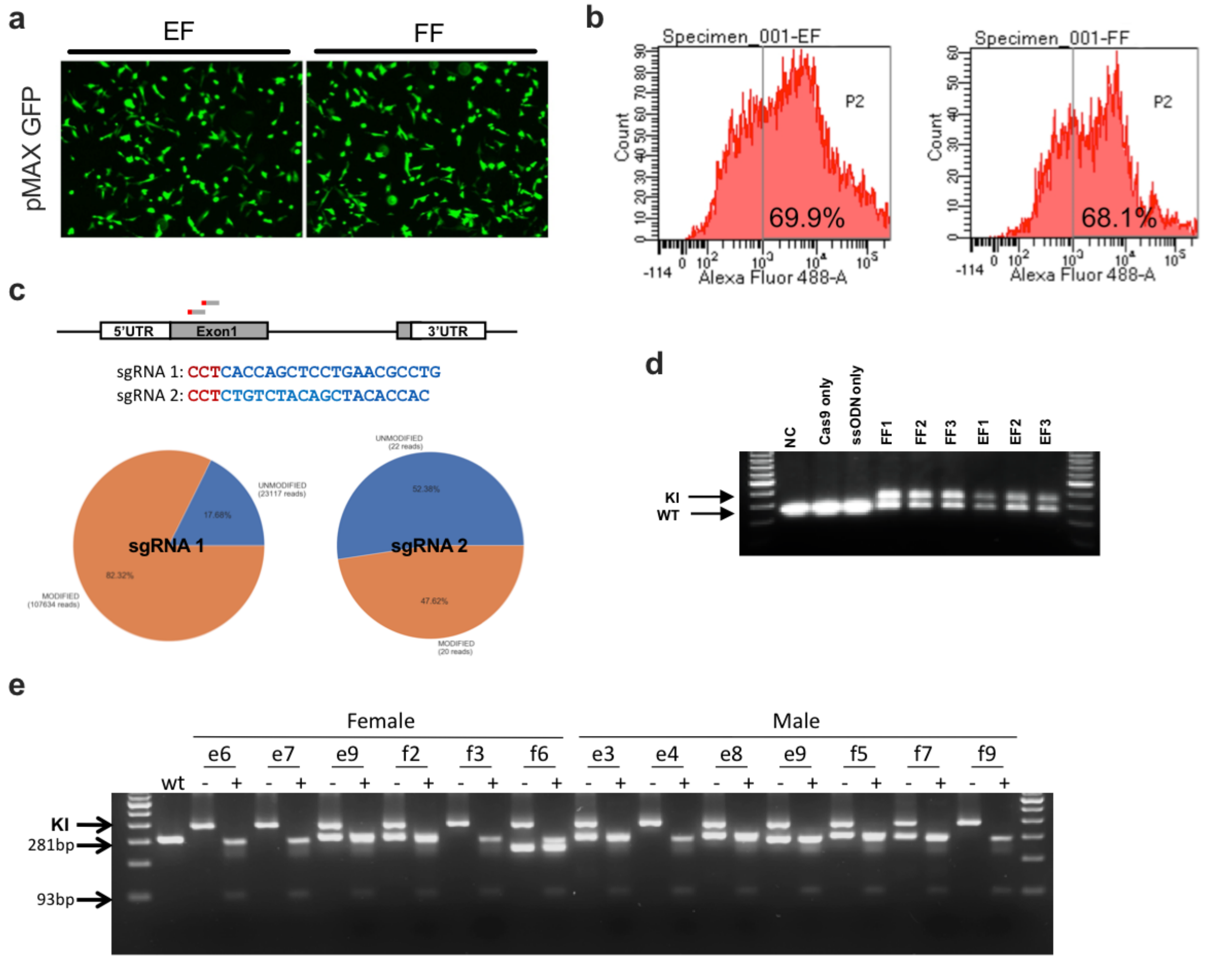

f

WT	GTGCCAGGACCAGAGGGTCAGAGGCCCAGCCCGGAATCCTCACCAGCTCCTGAACGCCTGTGCTCTTTCTGCAAACATAATGGCGA		
#1	GTGCCAGGACCAGAGGGTCAGAGGCCCAGCCCGGAATCCTCACCACAGCTCCTGAACGCCTGTGCTCTTTCTGCAAACATAATGGCGA	+2bp/-35 bp/-26 bp *	Mosaic
#2	GTGCCAGGACCAGAGGGTCAGAGGCCCAGCCCGGAATCCTCACCAGCTCCTGAACGCCTGTGCTCTTTCTGCAAACATAATGGCGA	WT/WT	-
#3	GTGCCAGGACCAGAGGGTCAGAGGCCCAGCCCGGAATCCTCACCCAGCTCCTGAACGCCTGTGCTCTTTCTGCAAACATAATGGCGA	+1 bp/-3 bp/+4 bp *	Mosaic
#4	GTGCCAGGACCAGAGGGTCAGAGGCCCAGCCCGGAATC--------CTCCTGAACGCCTGTGCTCTTTCTGCAAACATAATGGCGA	-8 bp/-10 bp/-12 bp *	Mosaic
#5	GTGCCAGGACCAGAGGGTCAGAGGCCCAGCCCGGAA----------CTCCTGAACGCCTGTGCTCTTTCTGCAAACATAATGGCGA	-11 bp/WT	Hetero
#6	GTGCCAGGACCAGAGGGTCAGAGGCCCAGCCCGGAATCCTCACCCAGCTCCTGAACGCCTGTGCTCTTTCTGCAAACATAATGGCGA	+1 bp/WT	Hetero
#7	GTGCCAGGACCAGAGGGTCAGAGGCCCAGCCCGGAATCCTC-----------AACGCCTGTGCTCTTTCTGCAAACATAATGGCGA	-11 bp/-13 bp/+2 bp *	Mosaic
#8	GTGCCAGGACCAGAGGGTCAGAGGCCCAGCCCGGAATC------------------------CTCTTTCTGCAAACATAATGGCGA	-24 bp/-4 bp	Biallelic
#9	GTGCCAGGACCAGAGGGTCAGAGGCCCAGCCCGGAA-----------TCCTGAACGCCTGTGCTCTTTCTGCAAACATAATGGCGA	-11bp	Homo
#10	GTGCCAGGACCAGAGGGTCAGAGGCCCAGCCCGGAA--------------TGAACGCCTGTGCTCTTTCTGCAAACATAATGGCGA	-11bp/-3 bp/WT	Mosaic

Figure. S3 related to Fig.4 CRISPR/Cas9-mediated modification of NANOS3 in embryonic fibroblasts. (a) Photographs and (b) flow cytometry results of fluorescence analysis of GFP expression in the EF and FF after the transfection of pMAX GFP plasmid. Cells were analyzed for GFP expression by fluorescence microscope (20x) and flow cytometry 72 hr from transient transfection**.** (c) Pie chart representation of amplicon sequencing analysis. Quantification of editing frequency as determined by the percentage and number of sequence reads showing unmodified (blue) and modified alleles (orange). CRISPResso classifies any mutation as an NHEJ event. The efficiency of on-site editing with candidate guide#1 was significantly higher (82.32%) than by guide#2 (47.62%). (d) Precise edits in transfected cells. PCR products generated using primers flanking the target sites are shown in Fig. 4a. (e) PCR and restriction enzyme analysis of individual clones with SalI. PCR products before (-) and after digestion with the appropriate restriction enzyme (+) are shown. A control reaction using untransfected cells is shown in the rightmost lane. (f) Sanger sequencing of injected blastocysts shows a range of mutations at the target site with high mosaicism.

Supplementary sTable. 1 Primers and Oligonucleotides used.

Application	Sequence
Gene editing	sgRNA-1 5'-CAGGCGTTCAGGAGCTGGTG-3'
	sgRNA-2 5'-GTGGTGTAGCTGTAGACAG-3'
	ssODN CCAGGACCAGAGGGTCAGAGGCCCAGCCCGGAATCCTCACAGGTCGACTACAAAGACCATGACGGTGATTATAAAGATCATGACATCGATTACAAGGATGACGATGACAAGTAACAGCTCCTGAACGCCTGTGCTCTTTCTGCAAACATAATGG
Genotyping	Forward 5'-TTCAACCTGTGGACAGATTACC-3'
	Reverse 5'-AGCTGTAGACAGAGGTGTAGCC-3'
iSeq	Forward 5'-GTGGGGAAGAGGAACCTGAGAC-3'
	Reverse 5'-AGCTGTAGACAGAGGTGTAGCC-3'

Supplementary sTable. 2 Gene expression marker gene-set for Fibroblasts

Species	Official gene symbol	Product description
Mm Hs	COL6A2	collagen type VI alpha 2 chain
Mm Hs	VTN	vitronectin
Mm Hs	VIM	vimentin
Mm Hs	PDGFRB	platelet derived growth factor receptor beta
Mm Hs	LUM	lumican
Mm Hs	POSTN	periostin
Mm Hs	ASPN	asporin
Mm Hs	MFAP5	microfibril associated protein 5
Mm Hs	COL1A1	collagen type I alpha 1 chain
Mm Hs	IL1R1	interleukin 1 receptor type 1
Mm Hs	FAP	fibroblast activation protein alpha
Mm Hs	CELA1	chymotrypsin like elastase family member 1
Mm Hs	LOX	lysyl oxidase
Mm Hs	P4HA1	prolyl 4-hydroxylase subunit alpha 1
Mm Hs	UCP2	uncoupling protein 2
Mm Hs	CCR2	C-C motif chemokine receptor 2
Mm	MS4A4C	membrane-spanning 4-domains, subfamily A, member 4C
Mm Hs	ITGAL	integrin subunit alpha L
Mm	EAR2	eosinophil-associated, ribonuclease A family, member 2
Mm Hs	FGR	FGR proto-oncogene, Src family tyrosine kinase

Mm Hs	HCK	HCK proto-oncogene, Src family tyrosine kinase
Mm	ADGRE4	adhesion G protein-coupled receptor E4
Mm Hs	TNFRSF1B	TNF receptor superfamily member 1B
Mm Hs	PRKCD	protein kinase C delta
Mm Hs	ENO3	enolase 3
Mm	CD209A	CD209a antigen
Mm Hs	ABI3	ABI family member 3
Mm Hs	TREML4	triggering receptor expressed on myeloid cells like 4
Mm	LY6I	lymphocyte antigen 6 complex, locus I
Mm Hs	PIP4K2A	phosphatidylinositol-5-phosphate 4-kinase type 2 alpha
Mm Hs	CD300E	CD300e molecule
Mm Hs	SERPINB10	serpin family B member 10
Mm Hs	CTHRC1	collagen triple helix repeat containing 1
Mm Hs	TBX18	T-box 18
Mm Hs	COL15A1	collagen type XV alpha 1 chain
Mm Hs	GJB2	gap junction protein beta 2
Mm Hs	IL34	interleukin 34
Mm Hs	EDN3	endothelin 3
Mm Hs	SLC6A13	solute carrier family 6 member 13
Mm Hs	ITIH5	inter-alpha-trypsin inhibitor heavy chain family member 5
Mm Hs	DPT	dermatopontin
Mm Hs	PENK	proenkephalin
Mm Hs	MMP14	matrix metallopeptidase 14
Mm Hs	ANGPTL2	angiopoietin like 2
Mm Hs	EFEMP1	EGF containing fibulin extracellular matrix protein 1
Mm Hs	SCARA5	scavenger receptor class A member 5
Mm Hs	IGFBP3	insulin like growth factor binding protein 3
Mm Hs	DPEP1	dipeptidase 1
Mm Hs	ADAMTS5	ADAM metallopeptidase with thrombospondin type 1 motif 5
Mm Hs	COL5A1	collagen type V alpha 1 chain
Mm Hs	CD248	CD248 molecule
Mm Hs	PI16	peptidase inhibitor 16
Mm Hs	PAMR1	peptidase domain containing associated with muscle regeneration 1
Mm Hs	TNXB	tenascin XB
Mm Hs	MMP2	matrix metallopeptidase 2
Mm Hs	COL14A1	collagen type XIV alpha 1 chain
Mm Hs	CLEC3B	C-type lectin domain family 3 member B
Mm Hs	IGFBP6	insulin like growth factor binding protein 6
Mm	AKR1C18	aldo-keto reductase family 1, member C18

Mm Hs	COL5A2	collagen type V alpha 2 chain
Mm Hs	FBN1	fibrillin 1
Mm Hs	FKBP10	FK506 binding protein 10
Mm Hs	PALLD	palladin, cytoskeletal associated protein
Mm Hs	WIF1	WNT inhibitory factor 1
Mm Hs	SNHG18	small nucleolar RNA host gene 18
Mm Hs	CDH11	cadherin 11
Mm Hs	PTCH1	patched 1
Mm Hs	ARAP1	ArfGAP with RhoGAP domain, ankyrin repeat and PH domain 1
Mm Hs	FBLN2	fibulin 2
Mm Hs	IGF1	insulin like growth factor 1
Mm Hs	PRRX1	paired related homeobox 1
Mm Hs	FKBP7	FK506 binding protein 7
Mm Hs	OAF	out at first homolog
Mm Hs	COL6A3	collagen type VI alpha 3 chain
Mm Hs	CTSK	cathepsin K
Mm Hs	DKK1	dickkopf WNT signaling pathway inhibitor 1
Hs	C1S	complement C1s
Mm Hs	RARRES2	retinoic acid receptor responder 2
Mm Hs	GREM1	gremlin 1, DAN family BMP antagonist
Mm Hs	SPON2	spondin 2
Mm Hs	TCF21	transcription factor 21
Mm Hs	PCSK6	proprotein convertase subtilisin/kexin type 6
Mm Hs	COL8A1	collagen type VIII alpha 1 chain
Mm Hs	ENTPD2	ectonucleoside triphosphate diphosphohydrolase 2
Mm	MMP23	matrix metallopeptidase 23
Hs	CXCL8	C-X-C motif chemokine ligand 8
Mm Hs	CXCL3	C-X-C motif chemokine ligand 3
Mm Hs	IL6	interleukin 6
Mm Hs	CYP1B1	cytochrome P450 family 1 subfamily B member 1
Mm Hs	COL13A1	collagen type XIII alpha 1 chain
Mm Hs	ADAMTS10	ADAM metallopeptidase with thrombospondin type 1 motif 10
Mm Hs	CCL11	C-C motif chemokine ligand 11
Mm Hs	ADAM33	ADAM metallopeptidase domain 33
Mm Hs	COL4A3	collagen type IV alpha 3 chain
Mm Hs	COL4A4	collagen type IV alpha 4 chain
Mm Hs	LAMA2	laminin subunit alpha 2
Mm Hs	ACKR3	atypical chemokine receptor 3
Mm Hs	CD55	CD55 molecule (Cromer blood group)

Mm Hs	FBLN7	fibulin 7
Mm Hs	FIBIN	fin bud initiation factor homolog (zebrafish)
Mm Hs	THBS2	thrombospondin 2
Mm Hs	NOV	nephroblastoma overexpressed
Mm Hs	PTX3	pentraxin 3
Mm Hs	MMP3	matrix metallopeptidase 3
Mm Hs	LRRK1	leucine rich repeat kinase 1
Mm Hs	HGF	hepatocyte growth factor
Mm Hs	FRZB	frizzled related protein
Mm Hs	COL12A1	collagen type XII alpha 1 chain
Mm Hs	COL7A1	collagen type VII alpha 1 chain
Mm Hs	MEOX1	mesenchyme homeobox 1
Mm Hs	PRG4	proteoglycan 4
Mm Hs	PKD2	polycystin 2, transient receptor potential cation channel
Mm Hs	CCL19	C-C motif chemokine ligand 19
Mm Hs	NNMT	nicotinamide N-methyltransferase
Mm Hs	FOXF1	forkhead box F1
Mm Hs	HAS1	hyaluronan synthase 1
Mm Hs	CTGF	connective tissue growth factor
Mm Hs	ERCC1	ERCC excision repair 1, endonuclease non-catalytic subunit
Mm Hs	WISP1	WNT1 inducible signaling pathway protein 1
Mm Hs	TWIST2	twist family bHLH transcription factor 2
Mm Hs	RIPK3	receptor interacting serine/threonine kinase 3
Mm Hs	DDR2	discoidin domain receptor tyrosine kinase 2
Mm Hs	ELN	elastin
Mm Hs	FN1	fibronectin 1
Mm Hs	HHIP	hedgehog interacting protein
Mm Hs	FMO2	flavin containing monooxygenase 2
Mm Hs	COL1A2	collagen type I alpha 2 chain
Mm Hs	COL3A1	collagen type III alpha 1 chain
Mm Hs	FSTL1	follistatin like 1
Mm Hs	GSN	gelsolin
Mm Hs	SPARC	secreted protein acidic and cysteine rich
Mm Hs	S100A4	S100 calcium binding protein A4
Mm Hs	NT5E	5'-nucleotidase ecto
Mm Hs	MGP	matrix Gla protein
Mm Hs	NOX4	NADPH oxidase 4
Mm Hs	THY1	Thy-1 cell surface antigen
Mm Hs	CD40	CD40 molecule

Mm Hs	SERPINH1	serpin family H member 1
Mm Hs	CD44	CD44 molecule (Indian blood group)
Mm Hs	PDGFRA	platelet derived growth factor receptor alpha
Mm Hs	EN1	engrailed homeobox 1
Mm Hs	DCN	decorin
Mm Hs	CEBPB	CCAAT enhancer binding protein beta
Mm Hs	EGR1	early growth response 1
Mm Hs	FOSL2	FOS like 2, AP-1 transcription factor subunit
Mm Hs	HIF1A	hypoxia inducible factor 1 subunit alpha
Mm Hs	KLF2	Kruppel like factor 2
Mm Hs	KLF4	Kruppel like factor 4
Mm Hs	KLF6	Kruppel like factor 6
Mm Hs	KLF9	Kruppel like factor 9
Mm Hs	NFAT5	nuclear factor of activated T cells 5
Mm Hs	NFATC1	nuclear factor of activated T cells 1
Mm Hs	NFKB1	nuclear factor kappa B subunit 1
Mm Hs	NR4A1	nuclear receptor subfamily 4 group A member 1
Mm Hs	NR4A2	nuclear receptor subfamily 4 group A member 2
Mm Hs	PBX1	PBX homeobox 1
Mm Hs	RUNX1	runt related transcription factor 1
Mm Hs	STAT3	signal transducer and activator of transcription 3
Mm Hs	TCF4	transcription factor 4
Mm Hs	ZEB2	zinc finger E-box binding homeobox 2
Mm Hs	LAMC1	laminin subunit gamma 1
Mm Hs	MEDAG	mesenteric estrogen dependent adipogenesis
Mm	MS4A4D	membrane-spanning 4-domains, subfamily A, member 4D
Mm Hs	LAMB1	laminin subunit beta 1
Mm Hs	DKK3	dickkopf WNT signaling pathway inhibitor 3
Mm Hs	TBX20	T-box 20
Mm Hs	MDK	midkine
Mm Hs	GSTM5	glutathione S-transferase mu 5
Mm Hs	NGF	nerve growth factor
Mm Hs	VEGFA	vascular endothelial growth factor A
Mm Hs	FGF2	fibroblast growth factor 2
Mm Hs	P4HTM	prolyl 4-hydroxylase, transmembrane
Mm Hs	CKAP4	cytoskeleton associated protein 4
Mm Hs	INMT	indolethylamine N-methyltransferase
Mm Hs	CXCL14	C-X-C motif chemokine ligand 14
Mm Hs	FLI1	Fli-1 proto-oncogene, ETS transcription factor

Mm Hs	FABP4	fatty acid binding protein 4
Mm Hs	COPZ2	coatomer protein complex subunit zeta 2
Mm Hs	FOSB	FosB proto-oncogene, AP-1 transcription factor subunit
